# Genome-edited *HEADING DATE* 3a knockout enhances leaf production in *Perilla frutescens*

**DOI:** 10.3389/fpls.2023.1133518

**Published:** 2023-04-03

**Authors:** Hee Rang Yun, Chong Chen, Jee Hye Kim, Hae Eun Kim, Sivabalan Karthik, Hye Jeong Kim, Young-Soo Chung, Hee Soon Baek, Sibum Sung, Hyun Uk Kim, Jae Bok Heo

**Affiliations:** ^1^ Department of Molecular Genetic Engineering, Dong-A University, Busan, Republic of Korea; ^2^ Crazy Peanut, lnc., Dong-A University, Busan, Republic of Korea; ^3^ Department of Molecular Biosciences and Institute for Cellular and Molecular Biology, University of Texas, Austin, TX, United States; ^4^ Department of Bioindustry and Bioresource Engineering, Sejong University, Seoul, Republic of Korea

**Keywords:** perilla, *FT*, *Hd3a*, flowering mechanism, CRISPR

## Abstract

Environmental cues regulate the transition of many plants from vegetative to flowering development. Day length, or photoperiod, is one cue that synchronizes flowering by changing seasons. Consequently, the molecular mechanism of flowering control is prominent in Arabidopsis and rice, where essential genes like *FLOWERING LOCUS* T (*FT*) homolog, *HEADING DATE* 3a (*Hd3a*), have been connected to flowering regulation. Perilla is a nutrient-rich leaf vegetable, and the flowering mechanism remains largely elusive. We identified flowering-related genes under short-day conditions using RNA sequencing to develop an enhanced leaf production trait using the flowering mechanism in the perilla. Initially, an *Hd3a*-like gene was cloned from the perilla and defined as *PfHd3a*. Furthermore, *PfHd3a* is highly rhythmically expressed in mature leaves under short-day and long-day conditions. Ectopic expression of *PfHd3a* in *Atft-1* mutant plants has been shown to complement Arabidopsis *FT* function, resulting in early flowering. In addition, our genetic approaches revealed that overexpression of *PfHd3a* in perilla caused early flowering. In contrast, the CRISPR/Cas9 generated *PfHd3a*-mutant perilla showed significantly late flowering, resulting in approximately 50% leaf production enhancement compared to the control. Our results suggest that *PfHd3a* plays a vital role in regulating flowering in the perilla and is a potential target for molecular breeding in the perilla.

## Introduction

*Perilla frutescens* is an annual herbaceous plant widely cultivated in Asian countries such as Korea, China, and India (Honda et al., 1990; Pandey and Bhatt, 2008). Perilla seeds contain approximately 45% oil, which is composed of over 90% unsaturated fatty acids such as oleic acid (18:1), linoleic acid (18:2), and linolenic acid (18:3) (Kim et al., 2019). Its leaves contain various functional compounds, including caffeic, rosmarinic, and γ-aminobutyric acids, as well as luteolin (Lee et al., 2009). Numerous studies have revealed that perilla is a valuable crop for culinary and pharmacological uses owing to its phytochemical content (Ahmed, 2018). Two perilla varieties, seed and vegetable, are extensively cultivated in East Asia (Choung, 2005). The seed variety is used as oil crops, whereas the vegetable variety is used as leafy crops for consumption or in Chinese medicine (Nitta et al., 2003). Choung (2005) reported that both perilla varieties showed differences in growth characteristics. In particular, the flowering date of seed varieties is approximately 23 days earlier than that of vegetable varieties, and the stem height and node numbers of seed varieties are higher than those of vegetable varieties (Choung, 2005). Regarding leaf characteristics, vegetable varieties’ leaf yield and cyanidin content are greater than those of seed varieties (Choung, 2005). However, the composition of fatty acids in seeds does not differ between the two varieties (Kim et al., 2019).

In Korea, the sowing season of perilla generally occurs in May, with leaf harvesting around the beginning of June to the end of September and seed harvesting from September to November (Gu et al., 2019). Perilla is a short-day (SD) plant, meaning flowering only occurs when the day length does not exceed a critical day length. Perilla becomes photosensitive at the fourth leaf pair stage; thus, it has been indicated that long nights tend to induce flowering (King and Zeevaart, 1973). Further, perilla floral stimulus movement and velocity are comparable to photosynthate, indicating the phloem transmits, and different varieties have different critical night length requirements for their flowering induction (King and Zeevaart, 1973). Perilla flowering usually starts 18–20 days after induction by long nights and blooms until it forms seeds after 30 long nights (King and Zeevaart, 1973). Recently, Kang et al. (2019) identified several genes through ortholog analysis, such as *GIGANTEA* (*GI*), *CONSTANS* (*CO*), and *EARLY FLOWERING 4* (*ELF4*), which are involved in the regulation of flowering time, suggesting that these putative perilla flowering orthologs are well conserved, as in other flowering plants. However, little is known about the molecular mechanisms underlying flowering in perilla.

Flowering plants have evolved mechanisms to control flowering time in response to environmental cues, including photoperiod, temperature, gibberellic acid, and ecological stresses; therefore, they coordinate flowering with particular seasons (Osnato et al., 2022). In Arabidopsis, several floral signaling pathways have been identified, and different types of flowering-regulation genes act in response to various factors and pathways (Amasino, 2010). These pathways converge on the floral integrator genes *FLOWERING LOCUS T* (*FT*), *SUPPRESSOR OF OVEREXPRESSION OF CONSTANS1* (*SOC1*), and *TWIN SISTER OF FT* (*TSF*) (Andrés and Coupland, 2012). The central genes that integrate multiple flowering signals in long-day (LD) and SD plants are *FT* and *FT* ortholog *Hd3a*, respectively (Kojima et al., 2002). Extensive studies on various flowering plants have shown that *FT* orthologs have been identified in other plants, such as peas, kiwifruit, tomato, rose, strawberry, and poplar (Pnueli et al., 2001; Böhlenius et al., 2006; Randoux et al., 2014; Sussmilch et al., 2015; Mouhu et al., 2009; Varkonyi-Gasic et al., 2013). Functional characterization of these genes revealed that most flowering plants have conserved molecular mechanisms of flowering.

This study aimed to (1) isolate a core gene of the flowering mechanism in perilla and characterize its functions in Arabidopsis and Perilla, (2) construct knock-out mutants using the CRISPR/Cas9 system to develop a new perilla trait for increasing leaf production. Hence, we characterized *PfHd3a*, a perilla *FT* ortholog, and its expression was specific under an SD photoperiod with a diurnal rhythmic pattern. Furthermore, complementation experiments showed that *PfHd3a* is the functional *FT* ortholog in perilla. Consistent with the previously described role of *FTs*, overexpressed P*fHd3a* causes early flowering in the perilla. In contrast, the *PfHd3a*-mutant perilla edited by a specific sgRNA flowered significantly later, increasing the perilla’s leaf production in genome-edited lines. Our results may lay a theoretical foundation for further enhanced leaf vegetable productivity development.

## Materials and methods

### Plant materials and growth conditions

The *Perilla frutescens* var. *japonica* HARA and cultivars Namcheon, Dayu, and Yeopsil were used in this study. Perilla seeds were surface sterilized with 50% Sodium Hypochlorite for 1.5 h, followed by 5 times wash with sterile distilled water and then sown on MS medium (Murashige and Skoog, 1962) supplemented with 0.8% sucrose, 0.6% agar, and pH5.7 plates. Seeds were grown at 22°C for LD conditions (PPFD-100 µmol m^-2^s^-1^;16h light/8h dark). To measure flowering days and qRT-PCR experiments, Namcheon perilla seeds and genetic resources #22 and #57 were sown in a seedling box and then transplanted in pots when two leaves were developed and grown on a growth chamber.

*Arabidopsis thaliana* (wild-type Col-0) and *ft-1* mutants were used in this study. Arabidopsis seeds were surface sterilized with 70% EtOH, washed 5 times with sterile distilled water, and then sown in MS medium plate. Seeds were grown at 22°C for 2 weeks, and then the seedlings were transplanted into sterilized soil and grown in a growth chamber for LD condition (PPFD-100 µmol m^-2^s^-1^;16h light/8h dark) and SD condition (PPFD-50 µmol m^-2^s^-1^;8h light/16h dark).

### Transcriptome *De novo* sequencing

An mRNA in total RNA was converted into a library of template molecules to make a cluster sequence using the Illumina TruSeq RNA Sample Preparation Kit (Illumina, Korea). Initially, poly-A-containing mRNA molecules were purified by poly-T oligo-attached magnetic beads. Following purification, the mRNA is fragmented into small pieces using divalent cations under elevated temperatures. The cleaved RNA fragments are copied into first-strand cDNA using reverse transcriptase and random hexamers.

This is followed by second-strand cDNA synthesis using DNA Polymerase I and RNase H. These cDNA fragments then go through an end repair process, adding a single ‘A’ base and ligating the adapters. The products are then purified and enriched with PCR to create the final cDNA library. Paired-end sequencing was performed using a NovaSeq 6000 platform (Illumina, Korea). This transcriptional dataset has been submitted to the NCBI (https://www.ncbi.nlm.nih.gov) and will be released with the reference PRJNA858612.

### Real-time PCR analysis

Perilla’ Namcheon’, ‘Dayu,’ and genetic resources #22 and #57 seeds were sown in pots and grown at 25°C under LD (16h light/8h dark) conditions in a growth chamber, and the upper layers, including leaves and stems, were cut and sampled when 3 true leaves were developed. For RNA analysis, total RNA from samples was extracted using NucleoZOL Reagent (MACHEREY-NAGEL, Germany). 5 µg of the total RNA was used for first strand cDNA synthesis using Oligo dT (Invitrogen, USA) Primer, and cDNA was synthesized according to the protocol of the SuperScript IV First-Strand Synthesis System kit (Invitrogen, USA). Quantitative real-time PCR containing specific primers was performed with the TOPrealTM qPCR 2X Premix (SYBR Green with low ROX). The sequence of each gene was obtained from RNA-sequencing data, and primers were prepared for the experiment (**Supplemental Table S2**).

### Over-expression plasmid construction

Initially, we performed complementation experiments on the *ft* mutant. Perilla cDNA was used to isolate the coding sequence (CDS) of the *PfHd3a* gene for constructing plants with over-expression of the *PfHd3a* gene. The obtained DNA fragments (525 bp) were inserted into the pDONR221 vector using BP clonase (Invitrogen, USA) based on the Gateway system and further moved into the pK7FWG2 and pB2GW7 vectors by LR clonase (Invitrogen, USA). All constructs were transformed into *Agrobacterium* GV3101 using the electroshock method and transformed into Arabidopsis wild-type and *ft* mutant plants according to the floral dip method.

### Transient expression assay

As Chen et al. (2020) described, a transient expression assay was performed. Overexpressed-PfHd3a transgenic plants were inoculated on MS medium and transplanted into the soil. After two weeks, we collected two primary leaves and thinly cut them with a blade before soaking them in 1M Mannitol for 30 minutes. After incubation, the sample was added to an enzyme solution (1% cellulase R-10, 0.25% mercerozyme R-10, MES, BSA, 500 mM Mannitol, 1mM CaCl_2_) and gently shaken before being incubated at 22°C for 12 hours in dark conditions. Subsequently, the enzyme solution was filtered through a 100-mm mesh. The protoplast solution is loaded onto 20 ml of 21% sucrose and centrifuged at 5000 rpm for 10 minutes. The intact protoplast was extracted and observed under a fluorescent microscope after centrifugation. The excitation filter detected the GFP signal in the range 470–550 nm (transmitting only green light).

### SgRNAs designed for CRISPR and plasmid construction

CRISPR/Cas9 reagents were cloned into the pBAtC binary vector having a whole CRISPR/Cas9 cassette to edit a target gene in perilla. Two sgRNAs (sgRNA1: TTACAAATGGCTGTGAATTT and sgRNA2: TACTGGAGCAACCTTTGGAC) were designed to recognize conserved regions in the coding sequence of the *PfHd3a* gene in perilla. Using Aar1 sites, both sgRNAs were ligated to the pBAtC vector and confirmed by sequencing. Constructs were transformed into *Agrobacterium* EHA105 using the electroshock method and transformed into perilla using plant tissue culture.

### Perilla transformation

Perilla transformations were performed as previously described (Lee et al., 2005). Perilla Yeopsil seeds were sown in MS medium (1x MS, 0.8% sucrose, and 0.8% agar, pH 5.7) in LD conditions (16h light/8h dark, 22°C). The cotyledons and hypocotyls were cut into 0.8~1cm size from 7 to 10 days old plants and immersed in *Agrobacterium* suspension for 30 minutes. Then samples were placed on sterilized filter paper to remove moisture and co-culture medium (1% MS medium, 3% Sucrose, 3mg/L 6-benzylaminopurine (BA), 0.1 mg L-1 α-Naphthaleneacetic (NAA), 1mM acetosyringone, 0.4% Gelrite) and cultured at 25°C under dark condition. After 3 days, the explants were washed 3 times using liquid MS medium and put on sterilized filter paper to remove moisture and cultured by adding on shoot induction medium (1% MS medium, 3% Sucrose, 3 mg L-1 BA, 0.1 mg L-1 NAA, 500 mg L-1 carbenicillin, 1.2 mg L-1 phosphinothricin (PPT), 0.4% Gelrite). After 6 to 10 weeks, the new shoots regenerated from the cotyledons, and hypocotyls were cut from the callus sections and put on the shoot elongation medium (1% MS medium, 3% Sucrose, 3 mg L-1 BA, 500 mg L-1 carbenicillin, 1.2 mg L-1 PPT, 0.4% Gelrite), then the roots are induced by putting it on the root induction medium (1% MS medium, 3% Sucrose, 0.4% Gelrite). After that, the transgenic plant was transferred to a pot, grown in a greenhouse, and the seeds were harvested to obtain transformed perilla seeds.

## Results

### *PfHd3a* is an *FT*-like gene expressed specifically under SD conditions in perilla

**Figure 1:**
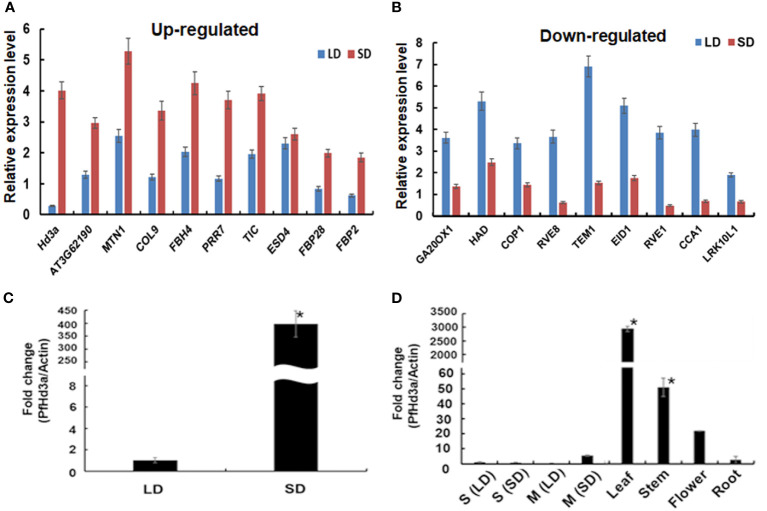
Transcript level of flowering related genes in perilla. **(A)** Transcript level of up-regulated and **(B)** down-regulated perilla flowering related genes under SD sample compared with LD sample. *De novo* RNA sequencing data were analyzed and transcript levels were compared between LD and SD sample. Average and error bar values were calculated with normalized read counts of two biological samples. Statistical significance was determined by Student’s test (*P < 0.05). **(C)** Quantitative transcript levels of *PfHd3a* gene under SD. Transcript levels of *PfHd3a* was compared by qRT-PCR between LD and SD. Statistical significance was determined by Student’s test (*P < 0.05). **(D)** *PfHd3a* expression in 2-week-old seedling (S), mature (M) perilla and various organs from one-month mature plants under SD condition as determined by qRT-PCR. LD: 16 h light/8 h dark. SD:8 h light/16 h dark. Statistical significance was determined by Student’s test (*P < 0.05).

A *de novo* transcriptome assembly of perilla was performed to generate a reference for high-throughput gene expression analysis and identification of critical flowering activator genes in the second leaf pair stage of young perilla plants grown under SD conditions. Differential expression of genes between perilla plants grown under SD and LD conditions was first analyzed using DEseq2 (Anders and Huber, 2010) with reference genes from the Ensembl Plants database (https://plants.ensembl.org/info/website/ftp/index.html). The raw data were then standardized and log2-transformed to form a scatter plot (**Supplemental Figure S1**). A total of 5,725 differentially expressed genes [DEGs; false discovery rate (FDR) ≤ 0.05; |fold-change (fc)| ≥ 2] were identified by comparing the two groups of samples (SD and LD). Significantly, 2,739 upregulated and 2,986 down regulated genes were counted by fold change (**Supplemental Figure S2**). We narrowed down the DEGs to select SD-specific flowering-related genes. We identified 18 novel perilla genes in the SD/LD comparison, of which 9 were upregulated and 9 were down regulated (**Figures 1A, B**). It was found that CONSTANS-like 9, AGAMOUS-like, and PSEUDO-RESPONSE REGULATOR 7 (PRR7) genes were upregulated, and many flowering-related genes such as *CONSTITUTIVE PHOTOMORPHOGENIC 1 (COP1), REVEILLE 1 (RVE1)*,and *CIRCADIAN CLOCK ASSOCIATED 1* (*CCA1*) were downregulated under SD conditions (**Figures 1A, B**). In particular, the *Hd3a*-like gene was highly expressed under SD conditions (**Figure 1A**), consistent with the levels of the *OsHd3a* gene, a major flowering activator in rice (Komiya et al., 2008). The perilla *Hd3a*-like gene was renamed *PfHd3a*. The expression of *PfHd3a* was validated using quantitative reverse transcriptase-polymerase chain reaction (qRT-PCR). The transcript level of *PfHd3a* in SD was > 300-fold higher than in LD (**Figure 1C**). In addition, *PfHd3a* was highly expressed in mature leaves but not in seedling plants (**Figure 1D**) by the expression of other *FT* homologs (Wickland and Hanzawa, 2015).

This suggests that *FT* functions as the main flowering activator in plants, and *FT* homologs are well conserved across many flowering plant species (Wickland and Hanzawa, 2015). To examine how widespread the *PfHd3a* protein is, a phylogenetic tree of *PfHd3a* was constructed using other plants. *PfHd3a* is highly homologous to other *FT* homologs, especially *Malus* x *demestica FT2* (MdFT2), *Beta vulgaris FT2* (BvFT2), and MdFT1 with 89%, 84%, and 84% identity, respectively (**Supplemental Figure S3**). In addition, *OsHd3a* and *AtFT* showed 82% and 80% amino acid sequence similarity to *PfHd3a*, respectively (**Supplemental Figure S3**). Notably, the amino acid alignment of *PfHd3a* with other *FT* homologs revealed that an external loop of protein sequences known as segment B, which is essential for *FT* function, was well conserved among *FT* homologs (**Supplemental Figure S4**, **S5**). It has also been reported that Tyr-85 and Gln-140 residues in segment B are critical for forming the ligand-binding pocket wall and are core residues for *FT* function (Laurie et al., 2011). Analysis of *PfHd3a* segment B showed that both residues were well conserved in the perilla homolog, similar to other *FT* homologs (**Supplemental Figure S5**), suggesting that *PfHd3a* may function as the effective flowering activator in perilla.

### *PfHd3a* is expressed in response to the photoperiod effect

**Figure 2:**
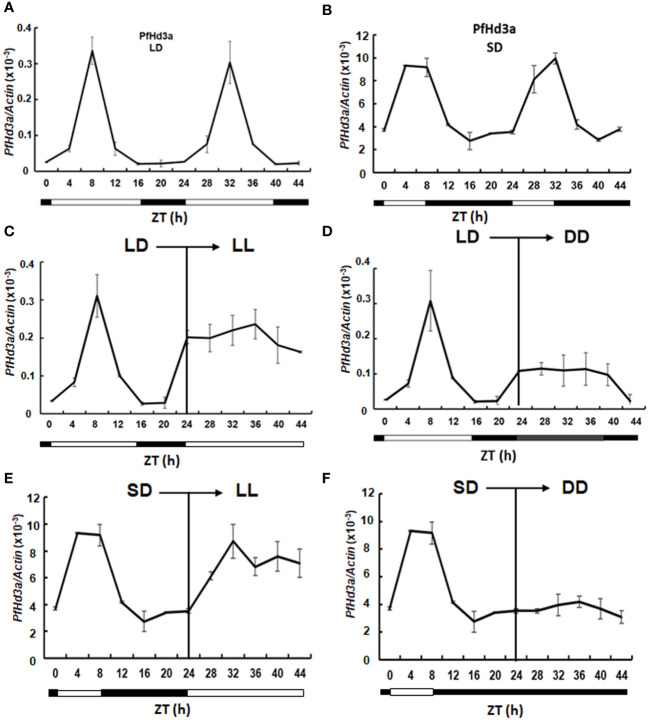
Expression analysis of *PfHd3a*. Rhythmic expression of PfHd3a under LD **(A)** and SD **(B)**. Expression analysis of PfHd3a under continuous light (LL) continuous dark (DD). The perillas were grown in growth chambers under LD (16 h light/8 darkness) and then transferred to LL **(C)** or DD **(D)** conditions. The perillas were grown in growth chambers under SD (8 h light/16 darkness) and then transferred to LL **(E)** or DD **(F)** conditions. White bars indicated light; black bars indicate darkness. The perilla actin gene was used as the internal control. Values represent means ± Standard deviation (SD) from two independent biological replicates.

Rice *Hd3a* and Arabidopsis *FT* transcript levels oscillate in distinct rhythmic patterns (Tsuji et al., 2015). We investigated the diurnal rhythm of expression of *PfHd3a* under SD and LD conditions by qRT-PCR. Interestingly, *PfHd3a* mRNA levels were diurnally regulated under SD and LD conditions. *PfHd3a* transcripts accumulated from the first appearance of light reached a peak at 8 h lights and then decreased until the end of the dark period (**Figures 2A, B**). To ascertain whether *the circadian clock-controlled PfHd3a transcript levels*, perilla grown under LD and SD conditions were transferred to continuous light or dark conditions, respectively, and *PfHd3a* transcript levels were verified. The rhythmic expression of *PfHd3a* quickly disappeared under continuous light and dark conditions (**Figures 2C, D**), indicating that *PfHd3a* is expressed rhythmically and is regulated by the photoperiod but not by the circadian clock.

### *PfHd3a* complements the Arabidopsis *FT* mutant

**Figure 3:**
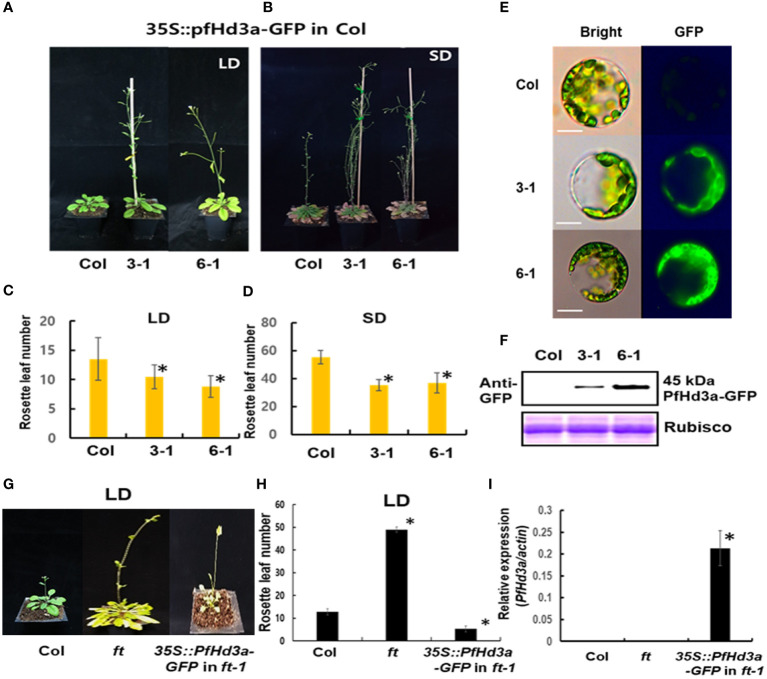
Flowering phenotype of overexpressed *PfHd3a* in *Arabidopsis*. Early flowering of 35S:*PfHd3a*-GFP in LD **(A)** and SD **(B)**. Flowering times were determined at total rosette leaf number at the time of flowering under LD **(C)** and SD **(D)**. At least 12 plants of each genotype were used. Statistical significance was determined by Student’s test (*P < 0.001) *versus* Col. **(E)** Arabidopsis mesophyll protoplasts were extracted from each genotype and the localization of PfHd3a-GFPs was observed by fluorescence microscopy. Scale bars = 10 μm. **(F)** Total proteins were extracted from Col and overexpression lines (3-1, 6-1) and then an immune blot was performed with an anti-GFP antibody. *Arabidopsis* Rubisco proteins were used as a loading control. **(G)** Complementation analysis of Arabidopsis *ft-1* mutant. *35S:PfHd3a-GFP* were transformed in *Arabidopsis ft* mutant and flowering phenotype was observed. **(H)** Flowering times were determined at the total rosette leaf number at the time of flowering under LD. At least 12 plants of each genotype were used. Statistical significance was determined by Student’s test (*P < 0.001). **(I)** The levels of *PfHd3a* mRNA under LD condition determined by qRT-PCR of Samples from 2-week-old plants. Each bar represents an average of two independent replicate experiments. The error bar indicates the standard deviation. Statistical significance was determined by Student’s test (*P < 0.05).

To assess the potential role of *PfHd3a* in flowering time regulation, the gene was overexpressed in Arabidopsis using the cauliflower mosaic virus 35S promoter. The *35S:PfHd3a* construct fused to green fluorescence protein (GFP) was transformed into Arabidopsis (Col) with *Agrobacterium tumefaciens*, and its flowering times were determined. At least 12 independents homozygous T3 lines were generated, and representative overexpressed-*PfHd3a*-GFP plants (III-1 and VI-1) showed early flowering under LD and SD conditions compared to Col plants (**Figures 3A–D**). *PfHd3a-*GFP protein expression was observed in protoplasts extracted from the overexpressing lines (**Figure 3E**). *PfHd3a-*GFP proteins were mainly expressed in the cytoplasm, as confirmed by western blotting using an anti-GFP antibody (**Figures 3E, F**). These results suggest that *PfHd3a* is involved in the regulation of flowering. To verify whether *PfHd3a* can complement the late-flowering of the Arabidopsis *ft-1* mutant, the same *35S:PfHd3a-GFP* construct was introduced into this mutant. As predicted, overexpressed-*PfHd3a* was able to fully complement Arabidopsis *FT* with rapid flowering compared to Col plants (**Figures 3G–I**). This adds to the findings that *PfHd3a* may act as a floral activator, similar to other *FT* orthologs.

### The *PfHd3a* transcript of seed perilla cultivars induces earlier flowering than that in vegetable perilla cultivars

**Figure 4:**
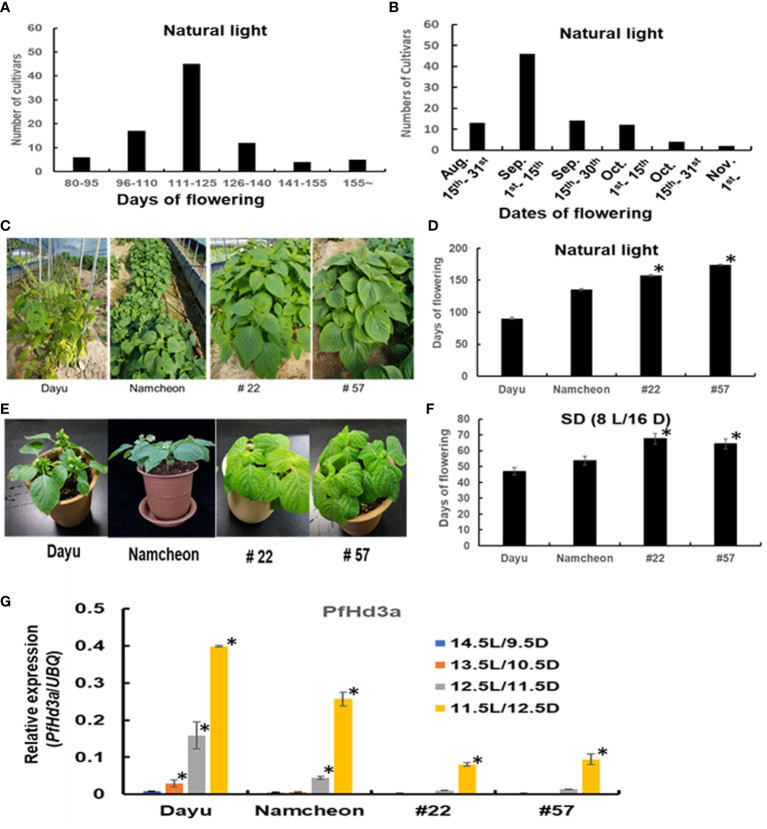
Characteristics of 90 perilla cultivars or perilla genetic resources. **(A)** The data represent frequency distributions of perilla cultivars based on days of flowering under natural light. **(B)** The data represent frequency distributions of perilla cultivars based on dates of flowering under natural light. **(C)** The flowering phenotype of seed perilla Dayu, vegetable perilla Namcheon and two perilla genetic resources #22 and #57 under natural light. **(D)** Flowering days of Dayu, Namcheon, #22, and #57 under natural light. The error bar indicates the standard deviation. Statistical significance was determined by Student’s test (*P < 0.05). **(E)** Flowering phenotype of Dayu, Namcheon, #22 and #57 under SD (8 h light/16 h darkness) condition. **(F)** Flowering days of Dayu, Namcheon, #22, and #57 under SD (8 h light/16 h darkness) condition. Statistical significance was determined by Student’s test (*P < 0.05). **(G)** The transcript levels of *PfHd3a* in Dayu, Namcheon, #22 and #57 under different light conditions. QRT-PCR was performed with cDNAs with 30-day-old perillas, dayu, namcheon, #22 and #57 grown in different light conditions. Statistical significance was determined by Student’s test (*P < 0.05).

As previously mentioned, flowering time is much earlier in seed varieties than in vegetable varieties. To evaluate the exact comparison of flowering time between seed perilla and vegetable perilla varieties, 90 perilla cultivars or perilla genetic resources were cultivated in the field, and their characteristics were analyzed (**Table 1**). The flowering of the perilla varieties ranged from 57–175 d after sowing (**Figure 4A**). For seed perilla planted on May 20th, the flowering period was usually from the beginning of August to the middle of September. In contrast, vegetable perilla varieties flowered from the beginning of October to the center of November (**Figure 4B**).

**Table 1:** Characteristics of 90 perilla cultivars or perilla genetic resources.

No.	Sowing date	Flowering date	No. of days to flowering	Flower color	Leaf color	No.	Sowing date	Flowering date	No. of days to flowering	Flower color	Leaf color
1	20-May	17-Aug	90	P	DG	46	20-May	30-Sep	134	W	G
2	20-May	03-Oct	137	W	G	47	20-May	23-Sep	127	P	DG
3	20-May	10-Sep	114	W	G	48	20-May	10-Nov	175	W	G
4	20-May	10-Sep	114	W	G	49	20-May	28-Aug	101	W	G
5	20-May	10-Sep	114	W	G	50	20-May	19-Aug	92	W	G
6	20-May	16-Sep	120	W	G	51	20-May	02-Sep	106	W	G
7	20-May	10-Sep	114	W	G	52	20-May	28-Sep	132	W	LG
8	20-May	10-Sep	114	W	G	53	20-May	10-Sep	114	W	G
9	20-May	10-Sep	114	W	G	54	20-May	10-Sep	114	W	G
10	20-May	06-Sep	110	W	G	55	20-May	16-Jul	58	W	G
11	20-May	19—Aug	92	W	G	56	20-May	22-Jul	64	W	LG
12	20-May	19-Aug	92	W	G	57	20-May	10-Nov	175	W	G
13	20-May	26-Aug	99	W	LG	58	20-May	03-Aug	76	W	LG
14	20-May	26-Aug	99	W	LG	59	20-May	14-Sep	118	P	GP
15	20-May	10-Sep	114	W	LG	60	20-May	10-Sep	114	W	G
16	20-May	06-Sep	110	W	LG	61	20-May	19-Aug	92	W	LG
17	20-May	10-Sep	114	W	LG	62	20-May	16-Sep	120	P	GP
18	20-May	10-Sep	114	W	G	63	20-May	16-Sep	120	W	G
19	20-May	10-Sep	114	W	LG, G	64	20-May	10-Sep	114	W	G
20	20-May	25-Oct	159	W	G	65	20-May	20-Aug	93	W	LG
21	20-May	15-Jul	57	W	LG	66	20-May	14-Oct	148	W	G
22	20-May	25-Oct	159	W	G	67	20-May	02-Sep	106	P	GP
23	20-May	10-Sep	114	W	G	68	20-May	10-Sep	114	P	GP
24	20-May	16-Sep	120	W	G	69	20-May	10-Sep	114	P	GP
25	20-May	25-Oct	159	W	DG	70	20-May	10-Sep	114	P	GP
26	20-May	02-Sep	106	W	G	71	20-May	28-Aug	101	W	G
27	20-May	10-Sep	114	P	GP	72	20-May	23-Sep	127	P	GP
28	20-May	06-Sep	110	P	GP	73	20-May	10-Sep	114	W	G
29	20-May	10-Sep	114	P	GP	74	20-May	10-Sep	114	W	LG
30	20-May	10-Sep	114	P	DG	75	20-May	10-Sep	114	W	LG
31	20-May	12-Sep	116	P	GP	76	20-May	06-Sep	110	W	LG
32	20-May	06-Sep	110	P	GP	77	20-May	06-Sep	110	W	G
33	20-May	02-Sep	106	P	GP	78	20-May	14-Sep	118	W	LG
34	20-May	20-Sep	124	P	GP	79	20-May	06-Sep	110	W	G
35	20-May	08-Oct	142	W	DG	80	20-May	10-Sep	114	P	GP
36	20-May	08-Oct	142	W	DG	81	20-May	16-Sep	120	W	G
37	20-May	16-Oct	150	P	G	82	20-May	10-Sep	114	W	G
38	20-May	08-Oct	139	E	DG	83	20-May	10-Sep	114	P	GP
39	20-May	20-Sep	124	W	G	84	20-May	07-Sep	111	P	DG
40	20-May	03-Oct	137	W	DG	85	20-May	07-Sep	111	P	DG
41	20-May	04-Oct	138	W	DG	86	20-May	07-Sep	111	P	DG
42	20-May	04-Oct	138	W	DG	87	20-May	22-Aug	95	P	GP
43	20-May	04-Oct	138	P	DG	88	20-May	08-Sep	112	P	GP
44	20-May	04-Oct	138	P	DG	89	20-May	08-Sep	112	P	GP
45	20-May	20-Sep	124	W	G	90	20-May	16-Sep	120	P	GP

The plants were cultivated in the field, and their flowering time, flower color and leaf color were analyzed. W, white; P, pink; E, etc.;

LG, light green; G, green; DG, dark green; GP, green purple; and P, purple.

The difference in flowering time between seed and vegetable perilla varieties prompted us to examine the induction time of *PfHd3a* transcripts under different day-length conditions. One seed variety, Dayu (#1), a vegetable variety, Namcheon (#40), and two perilla genetic resources, #22 and #57, which showed very late flowering, were chosen for this assay. First, when we examined the flowering time of the selected perilla under natural light, and SD (8L/16D) conditions; Dayu flowered rapidly under both conditions, followed by Namcheon, while #22 and #57 flowered late under both conditions (**Figures 4C–F**). To validate whether *PfHd3a* is involved in the different flowering times of these perilla varieties, the transcript levels of *PfHd3a* in each perilla were compared by qRT-PCR under different day-length conditions. *PfHd3a* transcript levels were deficient in all perilla under 14.5 L/9.5 D conditions, except for Dayu, which started increasing under 13.5 L/10.5 D conditions, and increased further with darker conditions (**Figure 4G**). In the case of Namcheon, *PfHd3a* transcript levels were detected from 12.5 L/11.5 D conditions, whereas *PfHd3a* levels appeared under 11.5 L/12.5 D conditions in #22 and #57 (**Figure 4G**). These results suggest that *PfHd3a* transcription is induced earlier in seed varieties compared to vegetable varieties and that the flowering time of seed perilla varieties is earlier than that of vegetable perilla varieties.

### *PfHd3a* is a flowering activator in perilla and the *PfHd3a* mutant enhances leaf production

**Figure 5:**
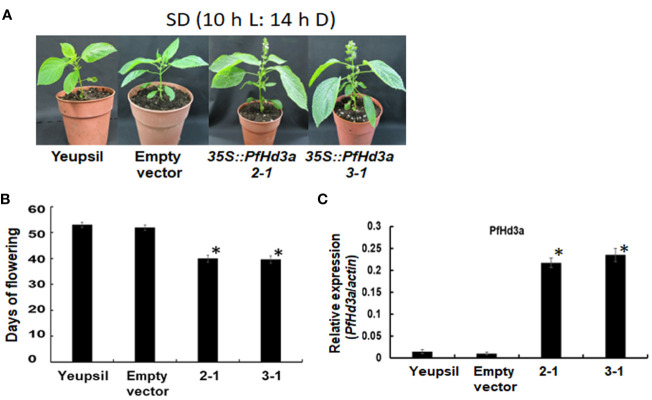
Flowering phenotype of transgenic perillas overexpressing *PfHd3a*. **(A)** Early flowering of 35S:*PfHd3a* 2-1 and 3-1 lines under SD (10 h light/14 h darkness) condition. **(B)** Flowering times were determined as days of flowering after sowing at the time of flowering under SD (10 h light/14 h darkness) condition. At least 12 plants of each genotype were used. Statistical significance was determined by Student’s test (*P < 0.001). **(C)** Transcript levels of *PfHd3a* in transgenic perillas. QRT-PCR was performed with cDNAs with 30-day-old yeopsil, and transgenic perillas (2-1 and 3-1) in SD (10 h light/14 h darkness) condition. Statistical significance was determined by Student’s test (*P < 0.05).

**Figure 6:**
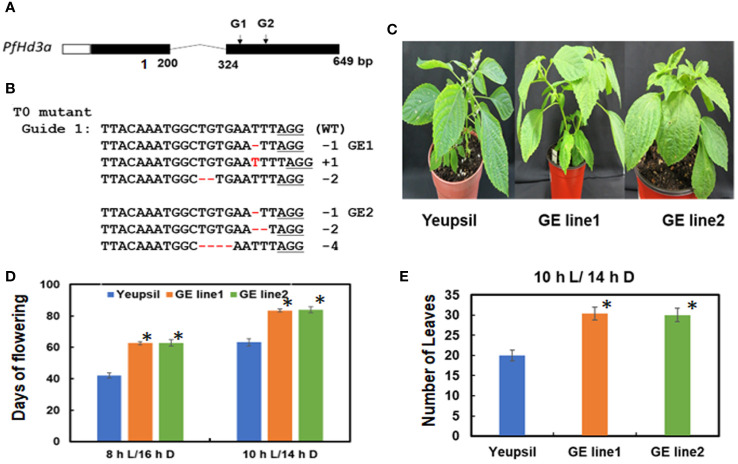
PfHd3a GE perillas enhance leaf productivity. **(A)** Schematic representation of *PfHd3*a locus. White, black boxes and curved lines represent 5’-untranslated regions (5’ UTR), coding sequence, and intron, respectively. **(B)** The aligned NGS reads flaking the *PfHd3a* target site and numbers represent the number of bases deleted and inserted in the T_0_ line. **(C)** The late-flowering phenotype of GE perillas. **(D)** Flowering times were determined as days of flowering after sowing at the time of flowering under SD (both 8 h light/16 h darkness and 10 h light/14 h darkness) conditions. At least 12 plants of each genotype were used. Statistical significance was determined by Student’s test (*P < 0.001). **(E)** Number of leaves were counted at the time of flowering under SD (10 h light/14 h darkness) condition. At least 12 plants of each genotype were used. Statistical significance was determined by Student’s test (*P < 0.001).

To further confirm the function of *PfHd3a* in perilla, a Korean perilla cultivar, Yeopsil (Lee et al., 2005), was transformed with the *35S:PfHd3a* construct. As a control, we transformed additional perilla plants with an empty vector and regenerated the perilla from cotyledon explants that had not been infected with *Agrobacterium tumefaciens.* Eleven independently generated *35S:PfHd3a* perilla was obtained, and the flowering time of T3 transgenic perilla (II-1 and III-1) was observed. Both transgenic perillas flowered significantly earlier than the regenerated control and parental perilla under SD conditions (10 L:14 D) (**Figures 5A, B**). When comparing the transcript levels of *PfHd3a* among transgenic and control perilla, approximately 11 and 13-fold higher levels were observed in the II-1 and III-1 lines, respectively (**Figure 5C**), reiterating that *PfHd3a* can induce flowering when overexpressed in perilla. To provide more direct evidence for the role of *PfHd3a* in flowering, we identified perilla plants carrying mutations in this gene. Gene-editing (GE) technology was employed using a reverse genetic approach. Two CRISPR-Cas9 target sites (guide (G) 1 and G2) were selected within the first and second exon of the *PfHd3a* gene (**Figure 6A**), and after preparing the oligo dimer, it was constructed into the CRISPR-Cas9 vector. Sequences were validated using U6 promoter primers. The correct construct was transformed in Yeopsil perilla using an *Agrobacterium*-mediated perilla transgenic method (Lee et al., 2005), and 10 positive plants were selected using phosphinotricin. To identify the mutation in the first and second exons of *PfHd3a* in transgenic lines, PCR amplification was performed, and the adjacent sequence of *PfHd3a* was sequenced in T0 generation plants. Five plants had mutations only in G1 of the first exon region. We measured the frequency of all mutant genotypes at the G1 target sites. We found that the main mutation types were single-base and amino acid frameshift mutations (**Figure 6B**). In the T2 generation, plants without exogenous DNA were selected. The flowering phenotype was observed under SD conditions (10 L/14 D). The gene-edited perillas showed significantly late flowering, resulting in approximately 50% leaf production enhancement compared to the control (**Figures 6C–E**), reiterating that *PfHd3a* is the main flowering activator in perilla.

## Discussion

Perilla plant varieties are cultivated in many Asian countries for multiple uses, such as curing depression-related diseases, anxiety, tumors, colds, fever, and chills (Ahmed, 2018). Various phytochemical compounds, specifically 271, have been isolated from perilla tissues and are expected to possess numerous health benefits for humans (Ahmed, 2018). Moreover, high levels of unsaturated fatty acids in perilla seeds reduce cholesterol and triglyceride levels in the serum (Ahmed, 2018). In addition, perilla has been used as a fresh edible aromatic vegetable plant to flavor foods. Due to the various perilla uses, it is a valuable crop to different Asian countries.

The flowering time for crops has a significant impact on production yield. In the case of leafy vegetables, including perilla, leaf production ceases following floral induction. Leafy vegetable perillas usually flower in the fall season, making it challenging to harvest perilla leaves as fresh vegetables in Korea around this time. The longest day of sunlight in Korea is June 21st, after which the daylight hours gradually decrease until December 22nd. Because fall and winter seasons are similar to short-day conditions which promote flowering, Korean farms usually illuminate their greenhouses to delay the flowering of cultivating perillas, allowing farmers to harvest perilla leaves well into the fall and winter seasons. Although illumination is helpful for leaf harvesting, the installation cost of light equipment in a greenhouse is an indispensable but costly consideration. Breeding is the best way to solve this issue, using perilla genetic resources from plant varieties that show delayed flowering, such as those referred to as #22 and #57. However, long-term breeding remains a significant challenge. Using biotechnological engineering, an alternative plan is manipulating flowering-related genes to develop late-bolting perilla. Therefore, this study identified many flowering-related genes in the perilla that could serve as genetic targets to prevent early flowering and allow for more vigorous harvests.

To date, several *FT*-like genes have been identified in various plants, and most of the identified *FT* homologs have been shown to play an essential role in floral transition (Turck et al., 2008). We characterized the *FT* homolog, *PfHd3a*, in perilla and examined the contribution of this gene to the control of flowering and photoperiod responsiveness. Our results revealed that the SD photoperiod strongly induces the expression of *PfHd3a* with a diurnal rhythm pattern (**Figures 1C**, **2**). Consistent with its role of promoting flowering under these conditions, *PfHd3a* could complement the *ft-1* allele when overexpressed in Arabidopsis (**Figure 3G**), and *PfHd3a* GE-mutant perillas flowered very late even under SD photoperiod conditions (**Figure 6C**). Therefore, we suggest that *PfHd3a* encodes a protein capable of activating flowering in perilla, similar to other *FT* homologs.

It is a possibility that the expression of *PfHd3a* is responsible, at least in part, for the differences in flowering time between seed and vegetable perilla varieties (**Figure 4G**). Under LD conditions (14.5L/9.5 D), *PfHd3a* transcripts were detected in only the seed perilla variety, Dayu, but not in the other varieties (**Figure 4G**). Under increased dark conditions (12.5L/11.5D) and (11.5L/12.5D), the expression of *PfHd3a* started increasing in vegetable perilla, Namcheon, and in both late-bolting perilla varieties, #22 and #57. These results raised another question as to why each perilla variety has a different expression time point of *PfHd3a*. Unquestionably, *PfHd3a* is involved in flowering time regulation in each perilla variety, but it is unclear whether each perilla variety has the exact up regulation mechanism for *PfHd3a* or not. One possible explanation for the differential expression time points may be the difference in the promoter region of *PfHd3a* in each perilla variety. In summer-annual accessions of *Arabidopsis*, up-regulation of *FT* under an LD photoperiod requires a long-distance enhancer in the promoter area to form a chromatin loop, resulting in high expression levels of *FT* (Tiwari et al., 2010; Cao Q et al., 2014). The seed varieties present a similar mechanism. Therefore, seed perilla varieties may have a different enhancer in the promoter region of *PfHd3a* from late-bolting varieties. This could be uncovered when genome sequencing of perilla is completed in the future.

Another possible explanation for the differential expression time points may be the differences in the epigenetic regulation of *PfHd3a* in different perilla varieties. In Arabidopsis, a core polycomb group repressive complex 2 (PRC2), CURLY LEAF (CLF), interacts with *FT* chromatin directly to catalyze H3K27me3 deposition (Cao S et al., 2014). CLF binding and H3K27me3 deposition at the *FT* locus antagonize nuclear factor (NF)-Y and CO binding, thereby inhibiting chromatin looping and *FT* expression in the late afternoon (Luo et al., 2018; Zhiguo et al., 2018). Similarly, a PRC1 consisting of EMBRYONIC FLOWER1 (EMF1), LIKE HETEROCHROMATIN PROTEIN (LHP1), and H3K4-demethylase Jumonji 14 (JMJ14), ensures repression of *FT* at night by binding to *FT* chromatin. Two additional EMF1-interacting H3K4 demethylases, JMJ15 and JMJ18, have also been shown to regulate polycomb group (PcG)-mediated *FT* repression (Feng and Lu, 2017; Yang et al., 2017). Several Jumonji-domain and CLF genes were identified in our RNA sequencing data (**Supplemental Table S1**), indicating that the PcG-mediated epigenetic regulation of *PfHd3a* may be well conserved in perillas. Therefore, it is possible that PcG-mediated *PfHd3a* repression may be vigorously implemented under LD photoperiods, and binding of the PcG-mediated repressive complex to *PfHd3a* chromatin may be disrupted under SD photoperiods in late-bolting perillas. This could repress the expression of *PfHd3a* under LD conditions and activate the expression of *PfHd3a* under SD conditions in late flowering perillas. However, there is a different regulatory mechanism of *PfHd3a* under LD and SD photoperiods between seed and vegetable perilla varieties. Nevertheless, we do not yet know which of these hypotheses is correct. Further studies are needed to address above issue shortly. Correspondingly, the accumulation of valuable medicinal and functional bioactive compounds decreases due to photoperiod sensitivity manipulation. We should also accomplish future research to quantify these bioactive substances in perilla.

The results described here demonstrate that *PfHd3a* promotes flowering under SD photoperiod conditions, and *PfHd3a* gene-edited mutant perilla delays flowering and produces more leaves under both LD and SD photoperiods. Given the sensitivity to photoperiod and late flowering phenotypes in perilla, the impairment of the *PfHd3a* gene increasing leaf production has been considered a promising strategy to maximize harvesting output. Although the phenotype of *PfHd3a* inactivation was determined, more studies are needed to evaluate the performance of these mutant lines. Hence, our findings would be incredibly significant for commercial agriculturists if they could break through the photoperiod sensitivity and produce numerous perilla leaves under long-day conditions.

## Data availability statement

The datasets presented in this study can be found in online repositories. The names of the repository/repositories and accession number(s) can be found below: https://www.ncbi.nlm.nih.gov/, PRJNA858612, ON952465.

## Author contributions

HY and CC conducted most of the experimentation. JK executed phenotype observations, measurements, and gene cloning. HEK examined *PfHd3a*-GFP in protoplasts. HJK, YC, and HB performed tissue culture of the perilla. SK, SS, HUK, and JH designed experiments and composed the manuscript. All authors contributed to the article and approved the submitted version.
