# Supplementary material for: Genome-edited HEADING DATE 3a knockout enhances leaf production in Perilla frutescens
Source: Front Plant Sci. 2023 Apr 3;14:1133518. doi: 10.3389/fpls.2023.1133518 (PMC10108627; doi:10.3389/fpls.2023.1133518)
Supplement: Supplementary file 1 [file DataSheet_1.pdf]

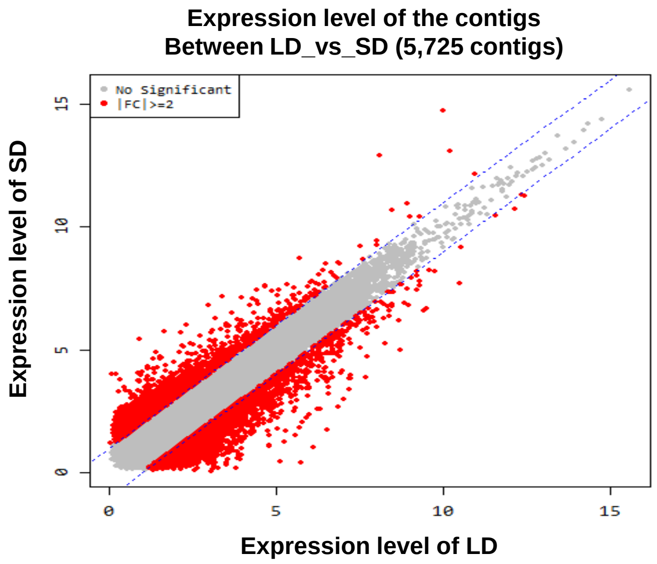

Figure S1 Transcriptome analysis isolating differentially expressed genes (DEGs) between LD and SD. Scatter plot of showing positive correlation between the transcriptome of perilla in LD and SD. Log2 fold change values of 52,212 active contigs in LD and SD samples were plotted in the x and y-axis, respectively. Gray dots are no significant different expression of genes. Red dots are more than two-foldup or down changed genes. Two biological replicates were used for the transcriptome analysis.

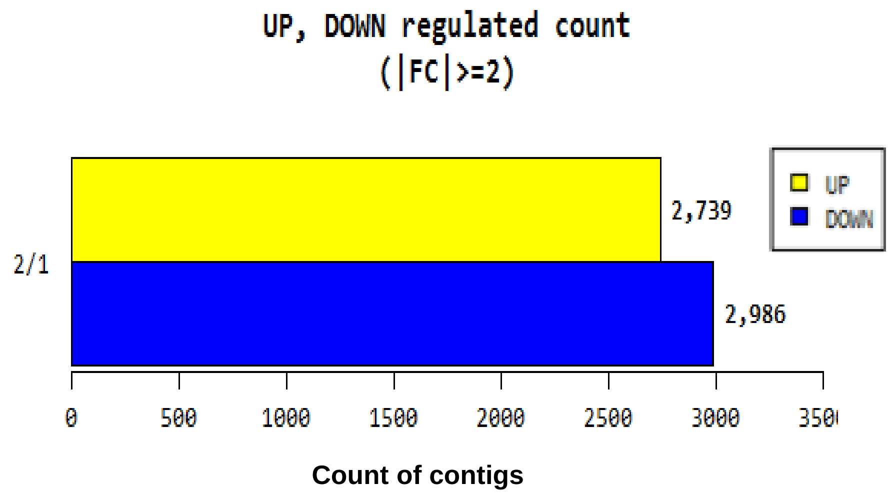

Figure S2 Significant up or down-regulated count by fold change. Bar showing total numbers of differentially up-regulated (yellow) and down-regulated (blue) genes under SD compared with the LD sample.

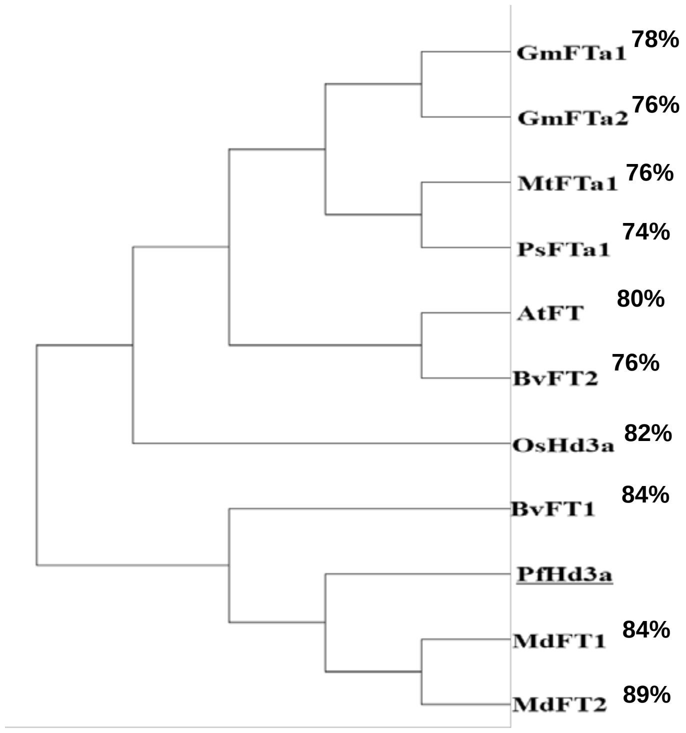

Figure S3 Sequence analysis of PfHd3a. Phylogenetic analysis of plant FT family proteins. The accession number of each gene is as follows: *PfHd3a* (*Perilla frutescens*, ON952465), *GmFTa1* (*Glycine max*, AB550124), *GmFTa2* (*Glycine max*, AB550125), *MtFTa1* (*Medicago*, HQ721813), *PsFTa1* (*Pisum sativum*, HQ538822), *AtFT* (Arabidopsis thaliana, AT1G65480), *BvFT2* (*Beta vulgaris*, HM448912), *OsHd3a* (Oryza sativa, AB052941), *BvFT1* (*Beta vulgaris*, HM448910), *MdFT1* (*Malus× domestica*, AB161112) and *MdFT2* (*Malus× domestica,* AB458504).

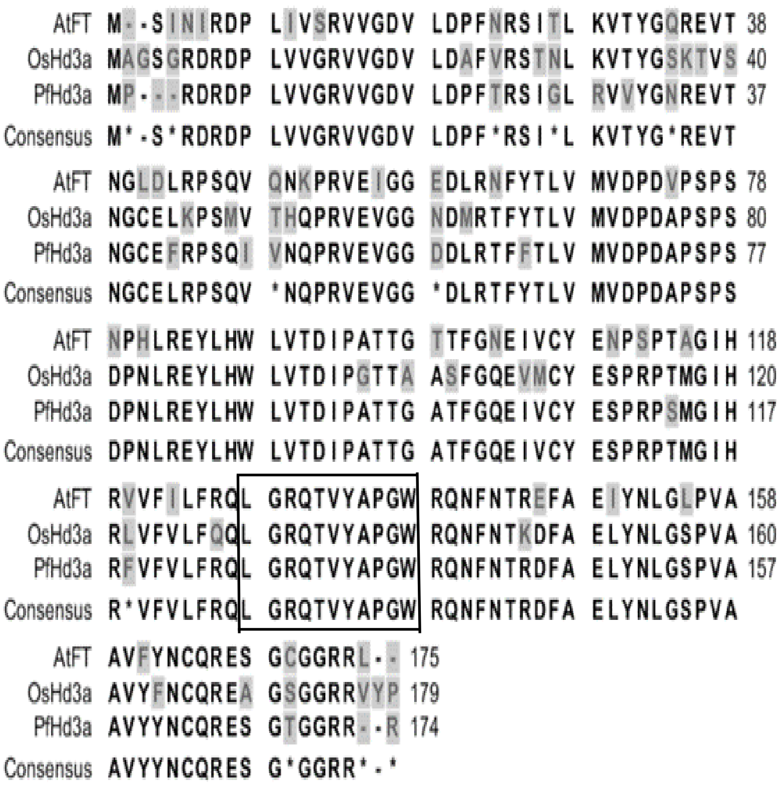

Figure S4 Amino acid sequence alignment of PfHd3a with AtFT and OsHd3a. Amino acid residues in gray represent unidentical amino acid and dots (*) indicates unconsensus amino acid. The black square indicates the conserved segmental region B.

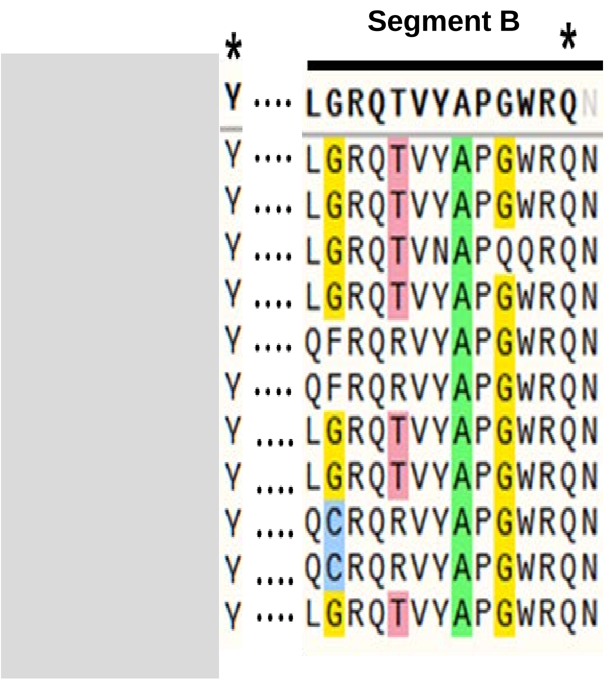

Figure S5 Corresponding to the external loop of the plant FT proteins. Sequences were aligned using ClustalW and analyzed with the Geneious software.
