# Supplementary material for: Genome-edited HEADING DATE 3a knockout enhances leaf production in Perilla frutescens
Source: Front Plant Sci. 2023 Apr 3;14:1133518. doi: 10.3389/fpls.2023.1133518 (PMC10108627; doi:10.3389/fpls.2023.1133518)
Supplement: Supplementary file 2 [file Presentation_1.pptx]

## Slide 1
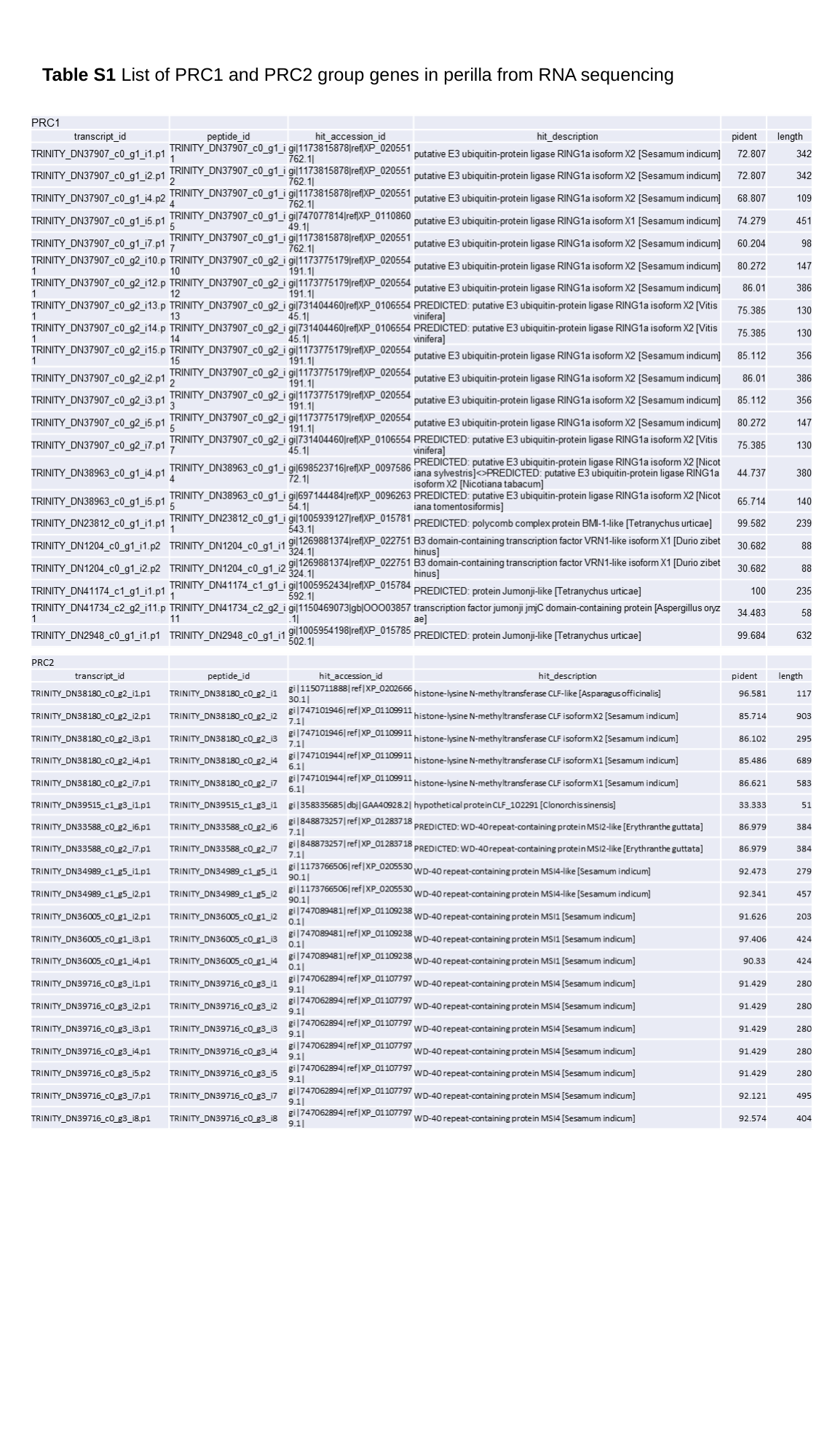

Table S1 List of PRC1 and PRC2 group genes in perilla from RNA sequencing

## Slide 2
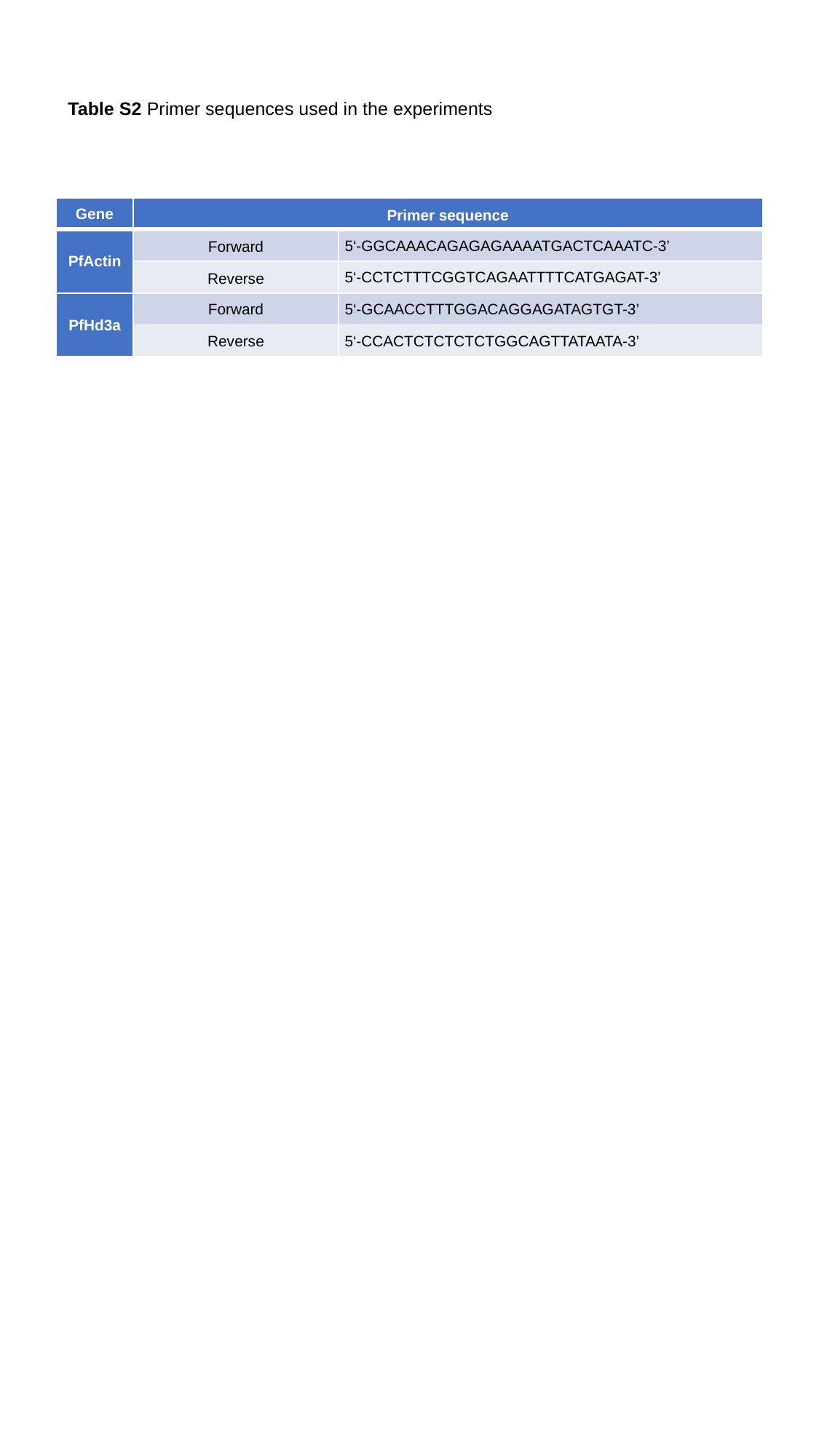

Table S2 Primer sequences used in the experiments
| Gene | Primer sequence | |
| --- | --- | --- |
| PfActin | Forward | 5‘-GGCAAACAGAGAGAAAATGACTCAAATC-3’ |
| | Reverse | 5‘-CCTCTTTCGGTCAGAATTTTCATGAGAT-3’ |
| PfHd3a | Forward | 5‘-GCAACCTTTGGACAGGAGATAGTGT-3’ |
| | Reverse | 5‘-CCACTCTCTCTCTGGCAGTTATAATA-3’ |
